# MORAL GAIN OR DECAY? EXAMINING AGE-RELATED CHANGES IN MORAL JUDGMENT

**DOI:** 10.1093/geroni/igz038.2888

**Published:** 2019-11-08

**Authors:** Minjie Lu, Helene H Fung

**Affiliations:** 1 Sun Yat-Sen University, Guangzhou, Guangdong, China; 2 The Chinese University of Hong kong, shatin, Not Applicable, Hong Kong

## Abstract

The present study investigates age-related changes in moral judgment. In particular, we examined both cognitive and affective dimensions of morality in contributing to moral punishment. One hundred and twenty participants (aged from 22 to 75) recruited from Mturk were presented with 10 moral transgression stories (e.g. lying, harming), and reported their wrongness judgment, moral conviction, emotional experience, and moral punishment. Results revealed divergent patterns on the relationships between age and the evaluations on cognition and emotion. In terms of cognitive evaluation, compared to younger adults, older adults perceived immoral acts as more wrong and considered their stands as more connected to their moral conviction. However, older adults reported less intense negative emotions (anger, disgust, contempt), suggesting they were less aroused by immoral acts. In terms of moral punishment, age was negatively correlated with punishment, and this correlation was mediated by the age-related decrease in negative emotions.

